# Foliar endophyte diversity in Eastern Asian-Eastern North American disjunct tree species – influences of host identity, environment, phylogeny, and geographic isolation

**DOI:** 10.3389/fpls.2023.1274746

**Published:** 2023-12-13

**Authors:** Wenbin Zhou, Wei Shi, Pamela S. Soltis, Douglas E. Soltis, Qiu-Yun (Jenny) Xiang

**Affiliations:** ^1^ Department of Plant and Microbial Biology, North Carolina State University, Raleigh, NC, United States; ^2^ Department of Crop and Soil Sciences, North Carolina State University, Raleigh, NC, United States; ^3^ Florida Museum of Natural History, University of Florida, Gainesville, FL, United States; ^4^ Department of Biology, University of Florida, Gainesville, FL, United States

**Keywords:** allopatric divergence, *Cornus*, comparative analyses, foliar endophytic community, fungal ITS, bacterial 16S rDNA, phylogeny, alpha and beta diversity

## Abstract

**Introduction:**

The well-known eastern Asian (EA) and eastern North American (ENA) floristic disjunction provides a unique system for biogeographic and evolutionary studies. Despite considerable interest in the disjunction, few studies have investigated the patterns and their underlying drivers of allopatric divergence in sister species or lineages isolated in the two areas. Endophyte diversity and assembly in disjunct sister taxa, as an ecological trait, may have played an important role in the processes of allopatric evolution, but no studies have examined endophytes in these lineages. Here we compared foliar endophytic fungi and bacteria-archaea (FEF and FEB) in 17 EA-ENA disjunct species or clade pairs from genera representing conifers and 10 orders of five major groups of angiosperms and 23 species of *Cornus* from EA and North America.

**Methods:**

Metagenomic sequencing of fungal ITS and bacterial-archaeal 16S rDNA was used to capture the foliar endophytic communities. Alpha and beta diversity of fungi and bacteria were compared at multiple scales and dimensions to gain insights into the relative roles of historical geographic isolation, host identity, phylogeny, and environment from samples at different sites in shaping endophytic diversity patterns.

**Results:**

We found that beta diversity of endophytes varied greatly among plant individuals within species and between species among genera at the same sampling site, and among three sampling sites, but little variation between region-of-origin of all plant species (EA vs ENA) and between EA-ENA disjunct counterparts within genera. Various numbers of indicator fungal species differing in abundance were identified for each plant genus and *Cornus* species. An overall significant correlation between endophyte community dissimilarity and phylogenetic distance of plants was detected among the disjunct genera but not among species of *Cornus*. However, significant correlations between beta diversities at different taxonomic scales of endophytes and phylogenetic distances of *Cornus* species were observed.

**Discussion:**

Our results suggest important roles of host identity and environment (sampling sites), and a likely minor role of phylogenetic divergence and historical biogeographic isolation in shaping the pattern of foliar endophyte diversity and assembly in the EA-ENA disjunct genera and *Cornus*. The results lead to a hypothesis that the sister taxa in EA and ENA likely differ in FEF and FEB when growing in native habitats due to differences in local environments, which may potentially drive allopatric divergence of the functional features of species.

## 1 Introduction

Deciduous forests in eastern Asia (EA) and eastern North America (ENA) contain numerous plant genera with sister species or clades (monophyletic groups of species) occurring disjunctly in the two areas (Li, 1952; Boufford and Spongberg, 1983; Wen, 1999). Many studies have been conducted to understand the phylogeny and biogeography of these disjunct genera to gain insights into the origin and evolution of the phytogeographic pattern (e.g., Xiang et al., 1998b; Xiang et al., 2000; Donoghue and Smith, 2004; Xiang et al., 2004; Harris et al., 2013; reviewed in Wen, 1999, and Wen et al., 2010; Wen et al., 2016). Recent studies have also examined morphological and molecular evolution in disjunct lineages, and some have integrated climatic data to decipher the underlying ecological drivers from a phylogenetic perspective (e.g., Du et al., 2020; Gaynor et al., 2020; Lindelof et al., 2020; Melton et al., 2020; Zhou et al., 2020; Melton et al., 2022; Qian et al., 2023). This large-scale phytogeographic pattern provides a unique opportunity for studying plant spatiotemporal evolution and the underlying mechanisms following allopatric speciation due to the presence of disjunct sister species or clades that have diverged over different lengths of time (Wen et al., 2010; Dong et al., 2019; Melton et al., 2020). For example, disjunct sister pairs are excellent for examining how genes and genomes might have diverged (e.g., Dong et al., 2019; Melton et al., 2020) and how morphology and ecology have differentiated over time following the historical geographic isolation (Du et al., 2020; Lindelof et al., 2020; Zhou et al., 2020; Melton et al., 2022). Nevertheless, few studies have focused on the endophytic aspect on the disjunct flora.

Foliar endophytes, as broadly defined, include fungi and prokaryotes that live within plant leaves (Petrini, 1991; Müller et al., 2015); they are known to not only affect plant phenotype and function but also may influence the assembly, diversity, and function of plant communities and entire ecosystems (Loreau et al., 2001; Balvanera et al., 2006; Ives and Carpenter, 2007; Van Der Heijden et al., 2008; Wani et al., 2015; Apigo and Oono, 2018; Griffin and Carson, 2018; Christian et al., 2020; Yang et al., 2019; Harrison and Griffin, 2020). However, our understanding of the functions and mechanisms of community assembly of these endophytes has been limited. Although some of these microbial endophytes inhabiting leaf tissues are known to have beneficial or harmful effects on their hosts, the functions of the majority of foliar endophytes have remained unknown and likely vary among taxa and host plants (reviewed by Rodriguez et al., 2009; McNees et al., 2019). There are a few hypotheses on mechanisms governing the assembly of endophyte communities (Van Bael et al., 2017): competition among endophytic species (Chase and Leibold, 2009), ecological drift/stochastic processes (Cordovez et al., 2019; Trivedi et al., 2020) and filters at various spatial and biological scales (Saunders et al., 2010). The filters include abiotic habitat filters, such as climatic and soil conditions, host-imposed biotic habitat filters such as plant traits, genotypes, and leaf phenology, and microbial species interactions (such as microbial competition, mutualism/facilitation, pathogen/parasitism, and predator/prey) (Saunders et al., 2010). However, the relative importance of these factors in a given lineage of host plants is largely unknown. The colonization of endophytes in foliar tissues can occur through vertical or horizontal transmission (Rodriguez et al., 2009; McNees et al., 2019). Vertical transmission involves the inheritance of fungi/bacteria from parent plants, which can be carried through seeds or pollen (Bright and Bulgheresi, 2010). Horizontal transmission occurs via airborne transmission, rain/flooding, litter sources, or infection through insect or herbivore attack, such as bacteria colonizing plant tissue through wounds or stomata (Bright and Bulgheresi, 2010; Bulgarelli et al., 2013; Christian et al., 2015). Therefore, the assembly of the foliar endophytic community depends on both transmission opportunities and subsequent interactions among the environment, microbial species, and host plants.

Given their potential functions, the endophyte communities in host plant species isolated in EA and ENA can therefore be viewed as an ecological feature that may play a key role in driving evolutionary divergence of morphology, speciation, and/or local adaptation of the disjunct taxa. However, no studies have compared endophytes among disjunct lineages to understand their variation pattern and decipher their potential roles in allopatric evolution of host species. Furthermore, despite empirical evidence on the importance of host plant community phylogeny and environment for structuring endophyte diversity and composition (Apigo and Oono, 2018), scant information is available on the assembly of endophytes, especially foliar microbiota, as affected by species-level phylogeny of host plants or by geographic isolation of sister lineages (Harrison and Griffin, 2020). A few surveys have revealed conflicts in terms of the relationship between plant phylogenetic distance and the community structure of foliar endophytic fungi (FEF, Vincent et al., 2016; Liu et al., 2019). For example, no significant correlation between host plant phylogenetic distance and the dissimilarity of FEF was drawn from a survey of 11 plant species belonging to five genera and five families (Vincent et al., 2016). However, by contrast, significant correlation was observed when examining 48 species of *Ficus* (Moraceae, Rosales, fabids, sensu Angiosperm Phylogeny Group IV, 2016) (Liu et al., 2019). Although geographic isolation is an important driver of speciation and a common mechanism generating biodiversity (reviewed in Coyne and Orr, 1998), its impact on the assembly of endophyte communities in diverging host species remains unknown. Therefore, species-level-phylogeny-based comparative studies of endophyte diversity for a diverse array of taxa disjunct between EA and ENA will be particularly useful to gain insights into the variation of endophyte communities between disjunct sisters and the role of phylogeny and historical biogeographic isolation on endophyte assembly.

In this study, we investigated foliar endophytes in 17 EA-ENA disjunct tree species pairs and 23 species of the dogwood genus *Cornus* L. (for a detailed investigation of a single plant genus). To seek answers for the following questions, our sampling strategy involved (1) trees of disjunct genera grown in a common arboretum, enabling us to eliminate the primary impact of local environment, and (2) multiple trees of the same species and genera grown in three common gardens in different geographic (or sampling) locations and climate to address the impact of environment. (1) Is there significant variation in foliar endophytic community structures between disjunct tree species pairs in all genera grown in the same environment? (2) Is the amount of difference in foliar endophytic community structure between a disjunct pair in a genus associated with the allopatric divergence of the disjunct pair? (3) Is there any “regional” difference of foliar endophyte diversity and composition when comparing all plant species from EA with all plant species from ENA grown at the same site? (4) Are there any foliar endophytes that are likely host-plant phylogenetic group-specific? (5) Is there correlation between foliar endophyte community dissimilarity and phylogenetic distance of host species across all disjunct genera? (6) What is the relative importance of host plant phylogenetic divergence, historical geographic isolation (i.e., region of origin), and the environments (i.e., sampling locations) on the foliar endophyte community? We sought to provide new insights into the assembly of foliar endophytes from the perspectives of host plant phylogeny and geographic isolation-based allopatric divergence.

## 2 Materials and methods

### 2.1 Sampling of host plants

**Figure 1:**
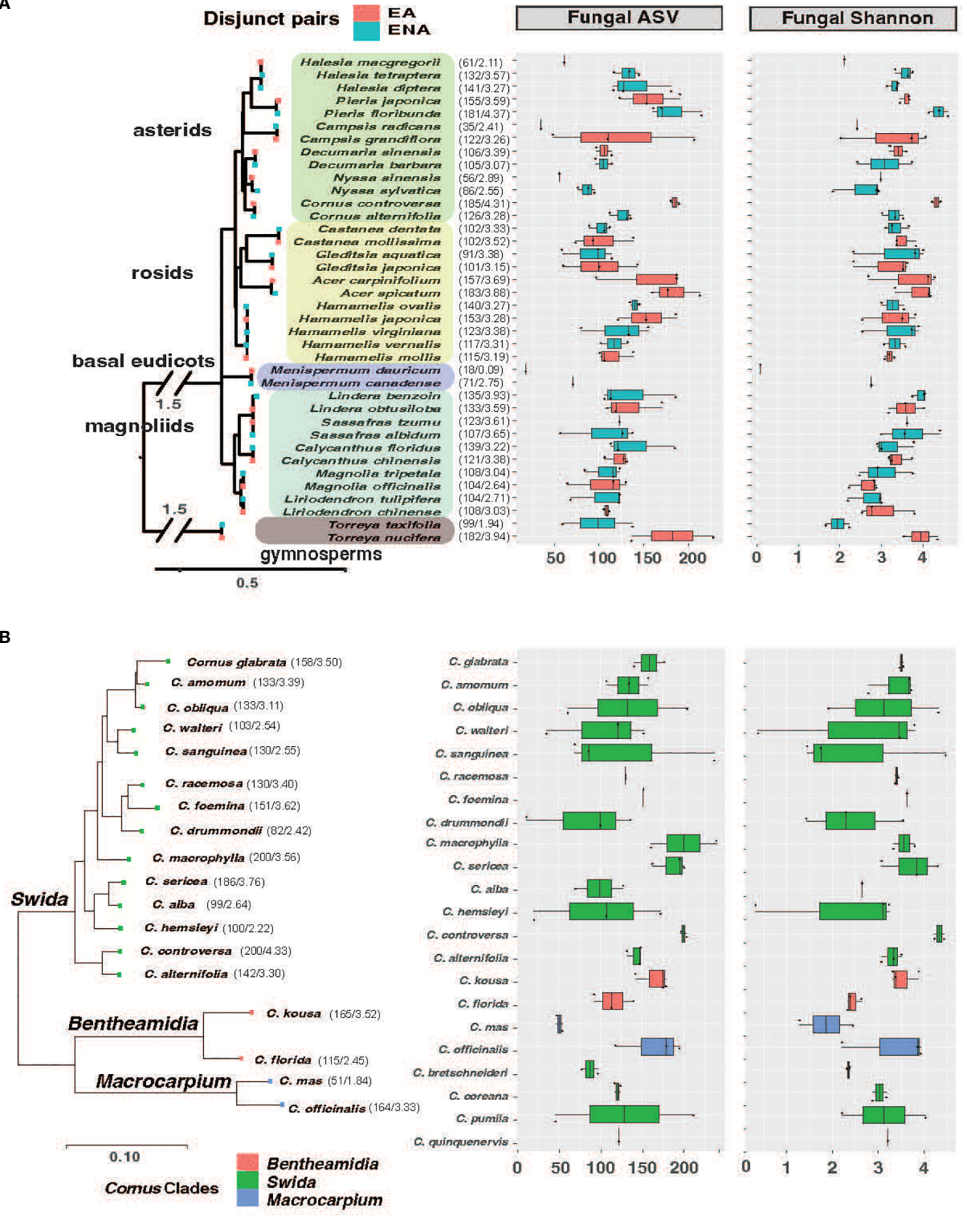
Phylogenetic tree of host species from EA-ENA disjunct pairs **(A)** and *Cornus* **(B)** combined with observed ASVs and Shannon index of fungal endophytes from each species. The phylogenetic tree of disjunct species was based on 14 shared chloroplast genes using the APG IV system (Angiosperm Phylogeny Group IV, 2016) as a backbone, while the phylogenetic tree of *Cornus* was based on 312 single copy nuclear genes from Hyb-Seq data (Thomas et al., 2021). The numbers after each species on the phylogeny represent the means of the ASVs and Shannon index.

Plant leaves were collected from the Arnold Arboretum in Boston, MA, USA, in July 2019, to examine how plant identities and their regions of origin affect the diversity and composition of leaf endophytes. This Arboretum has a large collection of EA-ENA disjunct plants and Cornaceae species. We sampled leaves from 101 accessions/individual plants from 38 species of 17 genera with species/clade pairs restricted to EA and ENA, representing one gymnosperm order (*Torreya*, Coniferales) and 10 angiosperm orders, encompassing magnoliids and multiple groups of eudicots: *Magnolia* and *Liriodendron* (Magnoliales, magnoliids), *Lindera*, *Sassafras*, and *Calycanthus* (Laurales, magnoliids), *Menispermum* (Ranunculales, “basal eudicot” grade), *Hamamelis* (Saxifragales, super-rosids), *Castanea* (Fagales, rosids), *Gleditsia* (Fabales, rosids), *Acer* (Sapindales, rosids), *Cornus*, *Decumaria*, and *Nyssa* (Cornales, asterids), *Halesia* and *Pieris* (Ericales, asterids), and *Campsis* (Lamiales, asterids) (**Supplementary Figure 1** showing GPS of sampled trees and dimension scale; **Supplementary Table 1**). In addition, we sampled 57 accessions representing 23 species of *Cornus* (**Supplementary Table 1**). For the downstream analyses, we classify them into five groups of disjunct genera based on angiosperm phylogeny (**Figure 1**; i.e., gymnosperms, magnoliids, basal eudicots, rosids, and asterids) and three clades of the dogwood genus based on dogwood phylogeny (**Figure 1**; i.e. the Big-Bracted dogwoods clade *Benthamidia*, the Blue- or White-fruited dogwoods clade *Swida*, and the Cornelian Cherries clade *Macrocarpium*).

We selected mature and healthy leaves (one to five leaves depending on the leaf size) from the lower canopy (around breast height) of the selected plants for sampling and collected leaf samples from one to three plants (used as replicates) for each species, depending on availability in the Arnold Arboretum in early July. Our sampling for the disjunct sister pairs included two species per genus except for *Halesia* and *Hamamelis.* For these two genera, we sampled three and five species, respectively. The species from each genus mostly represent the sister counterparts from EA and ENA with a single species from each region. In a few disjunct genera, there are two or more recognized species in one or both regions (e.g., *Acer, Pieris, Castanea*) (**Supplementary Table 1**). Fresh leaves were stored in a 4°C cooler and then transported to the lab within a day for cleaning and removal of microbes on the surface. Briefly, leaves were rinsed in 95% (vol/vol) ethanol for 30 sec, followed by a sequential immersion in 10% bleach with 0.05% Triton X-100 for 2 mins, 70% alcohol for 2 mins, and distilled water for 1 min (Greenfield et al., 2015; Wemheuer et al., 2019). Clean leaves were then dried in silica gel and stored in a -20°C freezer until DNA extraction.

To evaluate whether and how the same plant species grown in different geographic locations differ in their foliar endophytes, plant leaves were also collected from the J.C. Raulston Arboretum in Raleigh, NC, USA, Sarah P. Duke Gardens in Durham, NC, USA, and Jiangsu Botanical Garden in Nanjing, Jiangsu, China. Hereafter, these locations will be referred to as MA, NC, and Jiangsu. These samples help evaluate the relative role of plant host identity versus local environment in shaping the diversity and composition of leaf endophytes. To this end, 21 accessions of 12 species sampled at the Arnold Arboretum were obtained in NC (17 from the J. C. Raulston Arboretum and 4 from Sarah P. Duke Gardens), and 11 accessions of four species sampled at the Arnold Arboretum were obtained in China (**Supplementary Table 1**); these accessions were sampled and prepared by similar procedures as described above, except that the samples from China were dried in silica gel, stored at -20°C, and cleaned using 70% ethanol prior to DNA extraction.

### 2.2 DNA extraction and endophytic community library preparation and sequencing

Total DNA was extracted from leaf samples (~400 mg leaf materials) by the CTAB method of (Doyle, 1991) with modifications (Cullings, 1992; Xiang et al., 1998a). Then, ITS (fungi) and 16S rDNA (Bacteria/Archaea) libraries were prepared with Illumina adapter-appended primer pairs targeting the ITS1 region (F_KYO2: 5’- TAGAGGAAGTAAAAGTCGTAA-3’; R_KYO2: 5’-TTYRCTRCGTTCTTCATC-3’) and the V5-V6 region (799F: AACMGGATTAGATACCCKG; 1115R: 5’-AGGGTTGCGCTCGTTG-3’, which excludes chloroplast genes), respectively (Toju et al., 2012; Kembel et al., 2014), following the Metagenomic Sequencing Library Preparation protocol (Illumina, CA, USA). PCR was performed in a 50-μL solution containing 25 μL 2x KAPA HiFi HotStart ReadyMix (KAPA Biosystems, Wilmington, MA, USA), 2.5 μL template DNA (4-20 ng/μL), 2.5 μL 10 μM of each primer, and 17.5 μL nuclease-free water and under thermocycling conditions of initial denaturation at 95°C for 3 min, 25 cycles of 95°C for 30 sec, 55°C for 30 sec, and 72°C for 30 sec, and final elongation at 72°C for 5 min for both ITS and 16S rDNA [following the protocol from Illumina (2013)]. A negative control with no DNA template was also included to check for DNA contamination. PCR amplicons were cleaned with AMPure XP beads (Beckman Coulter Genomics, Danvers, MA, USA) and eluted in a 10 mM Tris buffer (pH 8.5). Purified PCR amplicons were then barcoded at both ends using the Nextera XT Index Kit (Illumina, San Diego, CA, USA) through 8 cycles of PCR with the aforementioned conditions and subsequently cleaned using the AMPure XP beads (Illumina, 2013). Both ITS and 16S rDNA amplicons were visualized on 1% agarose gels for quality checking, and the concentrations were calculated by Quant-iT PicoGreen dsDNA Assay kit (ThermoFisher Scientific, Waltham, MA, USA) using a synergy H1 Hybrid Reader (BioTek, Winooski, VT, USA). Then all samples were pooled in equimolarity for sequencing at the Genomic Science Lab, North Carolina State University (2 x 300 bp, v3 chemistry, Illumina, San Diego, CA, USA). All reads were deposited in the NCBI Sequence Read Archive (SRA) with the Bioproject accession number PRJNA772004.

### 2.3 Data analysis

#### 2.3.1 Endophytic diversity estimation and comparison

Demultiplexed raw reads were processed for removal of primers and adapters, filtration of spurious reads, and/or trimming to a fixed length using cutadapt v1.18 (Martin, 2011) and DADA2 (Callahan et al., 2016). Following deduplication, error model training, merging of paired-end reads, and chimera removal, a table of amplicon sequence variants (ASVs) and their copy numbers was generated. To retain most individual plants and to reflect the reliable diversity of the endophyte community after the removal of singletons, sequences were rarefied to 2,200, 4,000, and 5,000 for analyzing fungal diversity in leaf samples of disjunct species pairs from the Arnold Arboretum (MA), species of *Cornus* from the Arnold Arboretum, and duplicate plant species in three sampling locations (MA, NC, and Jiangsu, respectively). Sequences for bacterial/archaeal diversity were not rarefied because of the low abundance of amplicons in many individuals. For taxonomic classification, each ASV of fungi and Bacteria/Archaea was annotated according to the UNITE database v. 8.2 (Nilsson et al., 2019) and Greengenes database v. 13.8 (DeSantis et al., 2006), respectively, using Qiime2 (Caporaso et al., 2010; Bolyen et al., 2019).

The phyloseq package (McMurdie and Holmes, 2013) in R was used to estimate microbial alpha diversities (Observed ASV richness–ASVs and Shannon index) and beta diversity (Bray-Curtis dissimilarity, Bray and Curtis, 1957; Beals, 1984), which were visualized by principal coordinate analysis (PCoA). For the fungal endophytes, significant differences in alpha diversity, Shannon index, was assessed by non-parametric Kruskal-Wallis rank sum tests and the pairwise Wilcoxon rank sum test with false discovery rate adjusted using the Benjamini-Hochberg correction (Benjamini and Hochberg, 1995), while the observed number of ASVs was analyzed by ANOVA assuming a normal distribution. For the bacterial endophytes, all alpha diversities were assessed by non-parametric Kruskal-Wallis rank sum tests. We tested for significant differences in beta diversity by non-parametric permutational multivariate analysis of variance (PERMANOVA, Anderson, 2001; Anderson, 2014) and analysis of dissimilarities (ANOSIM, Anderson and Walsh, 2013) using the vegan package in R (Dixon, 2003). The tests were performed for the following: (1) between EA and ENA (i.e., all individuals and species tested by origin), (2) among the 17 disjunct genera, (3) among the five groups of disjunct genera (i.e., gymnosperms, magnoliids, basal eudicots, rosids, and asterids), (4) among 38 disjunct species, (5) among three major clades of *Cornus* (i.e., *Benthamidia* clade, *Swida* clade, and *Macrocarpium* clade), (6) among different sampling locations of the same species, and (7) between disjunct species pairs in each of the 17 disjunct genera (ANOSIM only). Due to the low number of bacterial ASVs detected in many individuals (see Results) (we did not detect any Archaea taxa in the sequences), only ASVs and Shannon index were calculated as measures of alpha diversity, and the sixth analysis above was not applied for the foliar endophytic bacterial (FEB) community. For all analyses above, we did not pool the reads of any species together. We performed Indicspecies in R (Cáceres and Legendre, 2009) to determine differentially abundant FEF taxa at different taxonomic ranks (phylum, class, order, family, and genus) among host plant species, which were based on the average sequence abundance in each plant species (all replicates from the same species were combined). The analyses using the Indicspecies package involved multiple comparisons with P value adjusted using r.g. method.

#### 2.3.2 Assessing correlation between phylogenetic distance and edophytic dissimilarity

Correlations between microbial Bray-Curtis dissimilarity and phylogenetic distance in plant phylogeny were evaluated in a Mantel test using the Spearman’s rank method (ecodist package in R, Goslee and Urban, 2007). For disjunct plant species pairs in the Arnold Arboretum, a phylogenetic tree was constructed using sequences from 14 shared chloroplast DNA regions longer than 120 bp (*atpB, matK, ndhF, psbC-trnS, psbM-trnD, rbcL, rpl16, rpoC1, rps4, rps16, trnD-trnT, trnH-psbA, trnL, trnS-trnfM*), including DNA sequences of ten species directly from GenBank and DNA sequences of the other species extracted from whole plastid genomes (23 species) and partial plastid genomes from Hyb-Seq data of Zhou et al. (2022) (five species) (**Supplementary Table 1**). Construction of a nuclear phylogeny for the disjunct pairs was not feasible due to lack of single-copy gene data for some of the genera or species. For *Cornus* species, a phylogenetic tree was constructed using sequences of 312 nuclear genes of 18 *Cornus* species (Thomas et al., 2021; Du et al., 2023) from Hyb-Seq data based on the Angiosperms353 kit (**Supplementary Table 1**) because sequence data were not available for *C. australis, C. bretschneideri, C. coreana, C. pumila*, and *C. quinquenervis* (see **Supplementary Table 1**). These species were not included in the phylogenetic analysis or subsequent correlation analyses. We aligned each data set using MAFFT (Katoh and Standley, 2013) and performed phylogenetic analyses using maximum likelihood (ML) in RAxML v. 8 (Stamatakis, 2014) with 1000 bootstrap replicates under the GTR+G model. The aligned data matrices for the disjunct taxa and *Cornus* are available at Dryad (https://doi.org/10.5061/dryad.s7h44j1cf).

Because the disjunct plant species pairs from different genera only sparsely represent seed plant phylogeny, we implemented the angiosperm phylogeny (Angiosperm Phylogeny Group IV, 2016) backbone with gymnosperms as the sister of angiosperms as a topological constraint using the “-g” parameter in our phylogenetic analysis to avoid obtaining a tree with incorrect topology due to low taxon sampling of the phylogeny. The pairwise phylogenetic distance was estimated based on the path-based branch lengths on the phylogeny using the cophenetic method in *stats* package in R (Sneath and Sokal, 1973). The five *Cornus* species for which we were not able to obtain molecular data for the phylogenetic analyses (**Supplementary Table 1**) were excluded from the Mantel test of correlation between phylogenetic distance and dissimilarity of endophytic communities. To obtain a pairwise dissimilarity metric of endophytic communities between different host species (including all replicates/individuals), we used the average abundance metrics of endophytic ASVs for each host plant species and calculated the Bray-Curtis distance for the pairwise plant species according to the endophytic taxonomic rank (phylum, class, order, family, and genus) and overall dissimilarity according to OTUs (ASVs) using the distance method in phyloseq in R (McMurdie and Holmes, 2013). Next, we computed the relationship between dissimilarity of endophytic communities and the phylogenetic distance of the host plants using the Mantel test for both the dataset of disjunct pairs and *Cornus*.

To tease apart the influences of phylogeny at deep and shallow divergence levels, we also specifically assessed the relationship between divergence time of sister counterparts in EA and ENA within each genus (approximated as branch lengths, which are presumably positively correlated with divergence time) and the fungal community differences (beta diversity) of sister counterparts among disjunct genera using a linear regression in R. The dissimilarity metric was based on the average sequence abundance in each plant species (all replicates from the same species were combined).

## 3 Results

### 3.1 Foliar endophyte alpha diversity

Overall, we generated 2,451,302 ITS sequences of foliar endophytic fungi and 90,216 16S rDNA sequences of foliar endophytic bacteria from 192 plant samples (no Archaea were detected). These sequences were clustered into 8,431 fungal ASVs and 983 bacterial ASVs with cyanobacteria (for photosynthesis) and mitochondrial DNA (384 ASVs, 28.1%) excluded by manually checking the classification results. After rarefaction, however, 73.5% (141 out of 192 samples) of the leaf samples did not show bacterial endophytes. Therefore, the results for bacterial endophytes were based on a non-rarefaction analysis.

We detected 4086 ASVs, 2880 ASVs, and 3840 ASVs of fungi after rarefaction among the disjunct genera, among *Cornus* species, and among sampling locations, respectively. The numbers of fungal ASVs (the mean of sampled individuals from the same host plant species) varied among species and regions, while there were no significant region-of-origin differences in alpha diversity of fungi between host plants from EA and ENA, among all *Cornus* species examined, and among the three sampling regions while there were significant differences in alpha diversity among disjunct genera (**Figure 1**; **Table 1**; **Supplementary Figure 2**; **Supplementary Table 2**). Alpha diversity exhibited a complex pattern showing greater differences within some genera than between genera in some cases but the reverse for others (**Figure 1A**). In general, host plant species with greater fungal ASVs also have greater fungal Shannon Index values (**Figure 1A**; **Supplementary Table 2**).

**Table 1:** Summary statistics of alpha diversities (ASVs and Shannon Index) for 17 disjunct pairs, *Cornus* L. clades, and sampling locations.

Comparisons	Fungi		Bacteria	
	ASVs	Shannon	ASVs	Shannon
17 disjunct genera				
Minimum	18	0.09	0	0
Maximum	185	4.37	59	3.39
Median	116	3.29	2	0.56
Mean	117	3.19	6	0.83
CV (%)	32.6	23	177.9	117.9
*Cornus* L. clades				
Minimum	51	1.86	0	0
Maximum	200	4.33	53	3.37
Median	130	3.17	8	1.68
Mean	132	3.05	12	1.56
CV (%)	28.7	19.7	105.3	60.2
Sampling locations				
Minimum	38	2.4	0	0
Maximum	249	4.33	94	3.8
Median	134	3.38	2	0.56
Mean	140	3.37	9	0.91
CV (%)	27.2	13.1	198.2	124.2
P values				
EA vs ENA	0.757	0.811	0.728	0.482
17 disjunct genera	**0.006**	**0.014**	**<0.001**	**<0.001**
*Cornus* clades	0.836	0.797	0.081	0.068
Sampling locations	0.93	0.442	**<0.001**	**0.003**

P values < 0.050 are marked in bold. The P values of ASVs were calculated by ANOVA, while Shannon Indices were calculated by Krustal-Wallis tests.

Data sources of this table are from **Supplementary Table 2**.

Compared to the fungal community, the bacterial community has far fewer ASVs detected. A total of 353 ASVs of bacteria was found in the disjunct genera, 367 ASVs were found in *Cornus*, and 400 ASVs were found in the samples from different sampling locations. The mean number of ASVs ranged from 0 to 34 across disjunct taxa and from 0 to 30 in *Cornus* (**Supplementary Figure 3**; **Supplementary Table 2**). The ASVs and Shannon Indices of bacterial communities have marginally significant differences among the three major clades of *Cornus* (**Table 1**; **Supplementary Table 2**). Among three sampling regions, we detected an average of four bacterial ASVs in plant individuals grown in MA, six bacterial ASVs in plant individuals grown in NC, and 31 bacterial ASVs in plant individuals grown in Jiangsu, and the differences were significant among the three regions (P < 0.001, **Table 1**; **Supplementary Figure 3**; **Supplementary Table 2**). The Shannon Index was also significantly different among the three sampling regions, with China having the highest values (2.03; P = 0.003, **Table 1**; **Supplementary Table 2**). We were not able to compare the differences within each location because of the paucity of amplified bacterial data.

### 3.2 Foliar endophyte composition

The fungal ASVs combined across species from disjunct pairs belong to five phyla, 25 classes, and 85 orders, while the fungi ASVs from *Cornus* belong to six phyla, 25 classes, and 80 orders according to the UNITE fungi database v. 8.2 (Nilsson et al., 2019; Kõljalg et al., 2020). Both were dominated by the same two phyla (Ascomycota and Basidiomycota), the same three classes (Dothideomycetes, Sordariomycetes, and Agaricomycetes), and the same two orders (Capnodiales and Pleosporales). In total, we found 12 dominant fungal ASVs with > 1% abundance in disjunct pairs and 13 dominant fungal ASVs with >1% abundance in *Cornus* (**Supplementary Figure 4**). Results from analysis of indicator species (i.e., fungal ASVs having significantly greater abundance in a specific host plant than in other host plants) showed that the number of fungal indicator species ranged from 15 (in *Gleditsia* and *Decumaria*) to 216 (in *Pieris*) and from 34 (in *Cornus australis*) to 170 (in *Cornus controversa*) among 17 different disjunct genera and within *Cornus*, respectively (**Supplementary Table 3**).

According to the Genome Taxonomy Database (Parks et al., 2022), the ASVs of FEB combined across species from disjunct pairs were annotated to 11 phyla, 21 classes, and 36 orders and dominated by one phylum (Proteobacteria), three classes (Alphaproteobacteria, Betaproteobacteria, and Actinobacteria), and two orders (Rhizobiales and Burkholderiales) (**Supplementary Figure 5A**); the bacterial ASVs from *Cornus* belong to eight phyla, 17 classes, and 29 orders, which were dominated by two phyla (Proteobacteria and Tenericutes), two classes (Gammaproteobacteria and Mollicutes), and two orders (Enterobacteriales and Acholeplasmatales) (**Supplementary Figure 5B**). The bacterial ASVs from three sampling locations were classified into 9 phyla, 19 classes, and 34 orders, which were dominated by one phylum (Proteobacteria), one class (Alphaproteobacteria), and one order (Rhizobiales) (**Supplementary Figure 5C**), a subset of the classes and orders seen in the disjunct pairs.

Among all disjunct species pairs (38 species) grown in the Arnold Arboretum, 30 fungal ASVs were shared by at least 25 (~65%) of the 38 disjunct species, while only two bacterial species were shared by at least 10 (~26%) of the 38 disjunct species (**Supplementary Table 4**). Most shared fungi were from phylum Ascomycota, class Dothideomycetes, and orders Pleosporales and Capnodiales, while the two shared bacteria were from two different families in two different orders, two different classes, and phylum Proteobacteria (**Supplementary Table 4**). In 23 *Cornus* species from the Arnold Arboretum, 16 fungal species were shared in at least 18 (~78%) of the 23 *Cornus* species, while only three bacterial species were shared in at least 15 (~65%) of the 23 *Cornus* species (**Supplementary Table 4**). Most shared fungi were from phylum Ascomycota, class Dothideomycetes, and order Capnodiales, while shared bacteria were from phyla Proteobacteria, Firmicutes, and Bacteroidetes (**Supplementary Table 4**). All shared fungal and bacterial taxa are among the aforementioned dominant groups.

### 3.3 Foliar endophyte beta diversity

#### 3.3.1 Fungal communities

Pairwise beta diversity (Bray-Curtis dissimilarity) among all 38 disjunct species from the Arnold Arboretum and between genera and between species of *Cornus* were very high, ranging from approximately 0.43 to 0.99 and from 0.31 to 0.99, respectively. Similarly, the beta diversity is very high in all species between plants from different geographic (or sampling) locations (with values mostly above 0.7 and up to 0.99) (see supplementary tables in Dryad). For the beta diversity among different species (based on the mean of all individuals from each species), the beta diversity was 0.54 – 0.99 in disjunct species; 0.42 – 0.98 in *Cornus*; and 0.52 – 0.99 among different sampling locations. These results indicate that the fungal compositions of the endophyte communities are very different among species and among sampling locations. (See original tables of beta diversity in Dryad: https://doi.org/10.5061/dryad.s7h44j1cf).

**Figure 2:**
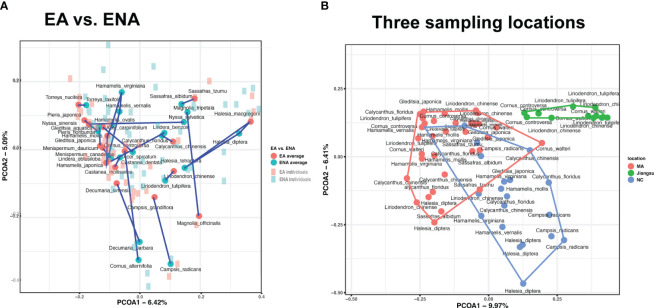
Principal-coordinate analyses (PCoA) of Bray-Curtis ITS profiles (beta-diversity of foliar fungal community) from **(A)** 17 disjunct pairs of EA-ENA counterparts and **(B)** three different geographical locations. Significant variations in plot **(A)** manifested mainly at the genus level (PERMANOVA, R^2 ^= 0.178, *P* = 0.001). Blue represents ENA, and red represent EA. Dark dots and light squares represents the coordination of each species and its individuals, respectively. Lines connect all species of the same genus. Variations in plot **(B)** were highly associated with sampling locations (PERMANOVA, R^2 ^= 0.124, *P* = 0.001).

PCoA showed no apparent separation pattern of foliar fungal endophytes (**Figure 2A**). Endophytes of congeneric plant species from EA and ENA (sampled from the Arnold Arboretum, MA) often did not cluster together (**Figure 2A**), but samples from ENA were more widely scattered in space than those from EA. Likewise, we found no phylogenetic group-specific clustering of host plants from the five phylogenetic groups examined (**Supplementary Figure 6A**). In contrast, the PCoA showed clustering of samples from the same sampling locations (**Figure 2B**). Samples from Jiangsu were well separated from samples from MA and NC, while samples from MA and NC were separated but somewhat overlapping (**Supplementary Figure 2C**).

Results of both the ANOSIM and PERMANOVA analyses indicated that the FEF assemblages (beta diversity) differed greatly among disjunct pairs/genera and geographic (or sampling) locations. The comparison among disjunct pairs/genera showed that only 1.4% (PERMANOVA test) of the variation was detected between species from EA and species from ENA, although this difference was significant. A total of 17.8% (PERMANOVA test) of the variation of FEF communities was attributed to variation among genera, 8.4% (PERMANOVA test) to variation among groups (i.e., gymnosperms, magnoliids, “basal” eudicots, rosids, and asterids), 22.3% (PERMANOVA test) to variation between species across all genera, and the remaining 50.0% (PERMANOVA test) to variation among individuals within species (**Table 2**). A separate comparison among sampling locations indicated that 12.4% (PERMANOVA test) of the variation of FEF communities was attributed to variation among sampling locations (**Table 2**). The beta diversity between all species from EA and all species from ENA was 0.53, lower than those values observed for individual species counterparts from the two regions (most above 0.7) (see Tables in Dryad). The ANOSIM results between EA-ENA disjunct pairs from each genus showed non-significant results for all genera, although results for pairs in *Cornus* (*P* = 0.09) and *Liriodendron* (*P* = 0.087) (**Supplementary Table 5**) were close to significant. This indicates that most interspecific variance of beta diversity is likely from species of different genera, and that differences between species of the same genus are not significantly greater than differences within species. It also indicates that a larger proportion of the variance of beta diversity within a genus came from variation among individuals within species (~50%) rather than from variation between species counterparts from EA vs. ENA (~22%) (PERMANOVA tests in **Table 2**).

**Table 2:** Comparisons of beta diversity of endophytic fungi and bacteria at different levels: among individuals within species, among species, among genera, among major clades, and among sampling locations for EA-ENA disjunct genera and *Cornus*.

Endophytes		ANOSIM		PERMANOVA	
	Comparisons	r	P	R^2^	P
Fungi	EA vs. ENA	**0.031**	**0.043**	**0.014**	**0.026**
	5 clades in disjunct taxa	**0.225**	**0.001**	**0.084**	**0.001**
	17 disjunct genera	**0.384**	**0.001**	**0.178**	**0.001**
	among species	**0.557**	**0.001**	**0.223**	**0.001**
	within species	NA	NA	0.501	NA
	3 *Cornus* major clades	0.030	0.386	**0.071**	**0.001**
	among *Cornus* species	**0.483**	**0.001**	**0.448**	**0.001**
	within *Cornus* species	NA	NA	0.481	NA
	3 sampling locations	**0.501**	**0.001**	**0.124**	**0.001**
Bacteria	EA vs. ENA	-0.012	0.689	**0.015**	**0.041**
	5 clades in disjunct taxa	**0.057**	**0.024**	**0.077**	**0.004**
	17 disjunct pairs	**0.188**	**0.001**	**0.225**	**0.001**
	among species	**0.456**	**0.001**	**0.273**	**0.001**
	within species	NA	NA	0.410	NA
	3 *Cornus* major clades	-0.213	0.984	**0.059**	**0.029**
	among *Cornus* species	**0.279**	**0.001**	**0.453**	**0.001**
	within *Cornus* species	NA	NA	0.488	NA
	3 sampling locations	**0.082**	**0.045**	**0.079**	**0.001**

A greater positive r value of ANOSIM indicates larger dissimilarity between groups, while a close-to-zero r value indicates less dissimilarity between groups; values of R^2^ in PERMANOVA indicate the percentages of variance explained by between groups; P-values in bold indicate significant differences.

NA means ‘Not applicable’.

PCoA showed no clustering of samples among the three *Cornus* clades (**Supplementary Figure 6B**). The ANOSIM and PERMANOVA results also indicated that only 7.1% (PERMANOVA test) of the variation of fungal endophyte communities occurred among the three major *Cornus* clades (although significant; *P* = 0.03 and 0.001, respectively), but 44.8% (PERMANOVA test) of the variation was attributed to variation between species within the genus, and the remaining 48.1% (PERMANOVA test) was attributed to variation within species (**Table 2**).

#### 3.3.2 Bacterial communities

The beta diversity (Bray-Curtis dissimilarity) of FEB between disjunct species, among *Cornus* species, and among different geographical regions ranged more widerly (0.14 - 1) than that of FEF communities. This is likely because the number of bacterial species from each individual plant was much smaller and varied more among plants and species compared to the fungal species.

PCoA of bacterial communities of disjunct genera showed an overall scattered pattern of samples (**Supplementary Figure 6**) except for the separation among samples collected from different sampling locations – MA, NC, and Jiangsu (**Supplementary Figure 6F**). However, the results of ANOSIM and PERMANOVA analyses showed a similar pattern of variation to FEF: only 1.5% of the variance was explained by EA vs. ENA host plants, while 7.7% of the variation was attributed to interspecific variation among the five phylogenetic groups of seed plants, and 22.5%, 27.3%, and 41.0% of the variation was explained by interspecific variation among genera, interspecific variation within genera, and intraspecific variation, respectively (PERMANOVA tests in **Table 2**). PCoA of the *Cornus* FEB community did not show significant differentiation among the three *Cornus* clades (**Supplementary Figure 6D**), but the results of ANOSIM and PERMANOVA analyses showed that 5.9% of the variation was among groups, while 45.3% of the variation was between species and 48.8% was within species (PERMANOVA tests in **Table 2**).

### 3.4 Relationship between phylogenetic distance and endophytic dissimilarity

**Figure 3:**
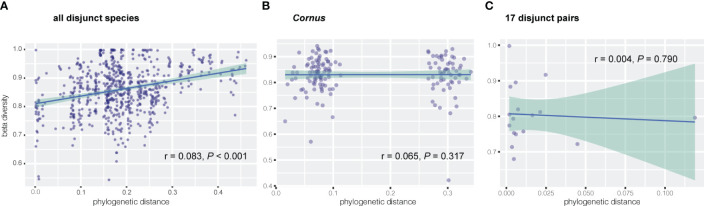
Correlation between dissimilarity of endophytic beta diversity and phylogenetic distance of host plant via Mantel test for **(A)** all disjunct species and **(B)** *Cornus* species, and via linear regression for **(C)** 17 disjunct pairs.

Results of Mantel tests showed that, overall, the phylogenetic distance of host plants was significantly correlated with fungal endophyte beta diversity (dissimilarity) among all species of the 17 disjunct genera (r = 0.083, P < 0.001; **Figure 3A**), but not correlated with the fungal endophyte diversity within *Cornus* (r = 0.065, *P* = 0.317; **Figure 3B**). Our results also showed that the phylogenetic distance of host species was significantly correlated with dissimilarity of three FEF taxa in disjunct pairs/genera and of six taxa within *Cornus* (**Table 3**).

**Table 3:** Mantel test between beta-diversity and phylogenetic distance using Spearman’s rank correlation.

Host plants	Endophytes Division	Specific endophytic taxa	r	P value
Disjunct	Class	p__Ascomycota|c__Eurotiomycetes	0.1445	0.068
		p__Ascomycota|c__Taphrinomycetes	0.1235	0.074
		p__Basidiomycota|c__Agaricomycetes	0.1665	0.051
		p__Basidiomycota|c__Cystobasidiomycetes	**0.2014**	**0.021**
	Order	p__Ascomycota|c__Eurotiomycetes|o__Chaetothyriales	0.161	0.064
		p__Ascomycota|c__Taphrinomycetes|o__Taphrinales	0.1235	0.072
		p__Basidiomycota|c__Agaricomycetes|o__Polyporales	**0.1641**	**0.042**
		p__Basidiomycota|c__Tremellomycetes|o__Tremellales	0.1259	0.09
	Family	p__Ascomycota|c__Sordariomycetes|o__Xylariales|f__Sporocadaceae	0.1454	0.09
		p__Ascomycota|c__Taphrinomycetes|o__Taphrinales|f__Taphrinaceae	0.123	0.087
	Genus	p__Ascomycota|c__Dothideomycetes|o__Pleosporales|f__Pleosporaceae|g__Alternaria	**0.1865**	**0.035**
		p__Ascomycota|c__Sordariomycetes|o__Xylariales|f__Sporocadaceae|g__Pestalotiopsis	0.1785	0.064
		p__Ascomycota|c__Taphrinomycetes|o__Taphrinales|f__Taphrinaceae|g__Taphrina	0.123	0.09
*Cornus*	Class	p__Ascomycota|c__Leotiomycetes	0.2672	0.056
	Order	p__Ascomycota|c__Leotiomycetes|o__Erysiphales	**0.3292**	**0.041**
		p__Ascomycota|c__Leotiomycetes|o__Helotiales	**0.4599**	**0.003**
	Family	p__Ascomycota|c__Leotiomycetes|o__Erysiphales|f__Erysiphaceae	**0.3292**	**0.035**
		p__Ascomycota|c__Sordariomycetes|o__Diaporthales|f__Gnomoniaceae	0.1963	0.1
	Genus	p__Ascomycota|c__Dothideomycetes|o__Capnodiales|f__Mycosphaerellaceae|g__Sphaerulina	**0.6004**	**0.002**
		p__Ascomycota|c__Dothideomycetes|o__Pleosporales|f__Pleosporaceae|g__Alternaria	0.1757	0.109
		p__Ascomycota|c__Leotiomycetes|o__Erysiphales|f__Erysiphaceae|g__Erysiphe	**0.3314**	**0.045**
		p__Ascomycota|c__Sordariomycetes|o__Diaporthales|f__Gnomoniaceae|g__Discula	**0.3948**	**0.039**

P-values (<0.05) in bold indicate significant correlation. These p-values were not corrected for multiple comparisons. Additional dedicated studies are needed to confirm the results.

Linear regression of FEF beta diversity on phylogenetic divergence of EA-ENA plant counterparts did not show a significant relationship between the divergence time (approximated as branch lengths) of sister species from EA and ENA and their difference in fungal communities (based on beta diversity) (r = 0.004, *P* = 0.790) (**Figure 3C**). Most disjunct species pairs had low phylogenetic divergence, but their beta diversity was high and varied among genera (See supplementary tables in Dryad: https://doi.org/10.5061/dryad.s7h44j1cf).

## 4 Discussion

How lineages differentiate following geographic isolation (regions of origin) in the absence of gene flow is an important question in evolutionary biology. The EA-ENA disjunct sister taxa in many plant genera provide unusual opportunities to gain insights into this question. Although numerous studies have been conducted on the disjunct taxa, how sister taxa isolated in EA and ENA have diverged over time still requires study. Foliar endophytic diversity and composition are important ecological properties of plants but have not been investigated for EA-ENA disjunct lineages. Our study is the first report of foliar endophyte diversity in EA-ENA disjunct genera and of patterns of variation of endophyte diversity across different scales, from within species to among genera from the same sampling location, among samples of the same taxa from different locations, and between the two geographic origins of species, EA and ENA.

Our results from analyses of a diverse array of samples from the same location indicated that a large amount of variation of foliar endophyte communities was from intraspecific variation (50.1% for fungi and 41.0% for bacteria), among species (22.3% for fungi and 22.5% for bacteria), and among genera (17.8% for fungi and 22.5% for bacteria) (R^2^ values from the PERMANOVA test in **Table 2**). However, neither the alpha nor beta diversities based on all individual data showed significant differences between disjunct species counterparts within most genera (**Figure 1A**; **Supplementary Table 5**) for samples grown in the same environment (i.e., the Arnold Arboretum), although their values of Bray–Curtis dissimilarity appeared to be high, ranging from 0.43 to 0.99. The data suggest that historical geographic isolation of the species pairs of most of the genera studied has not resulted in significant intrinsic divergence in their ability to host similar endophyte community assemblies, although they have been isolated for millions of years (see Wen et al., 2010).

Effects of environmental factors on FEF assemblages have been well-documented (e.g., Zimmerman and Vitousek, 2012; Whitaker et al., 2018). Our comparisons of duplicate host species from three different location sites similarly support a major role of the environment in FEF assemblages (**Figure 2B**; **Table 2**). For instance, we observed significant differences of FEF diversity and composition in the samples of the same host plant species from MA (USA), NC (USA), and Jiangsu (China) (**Figure 2B**; **Table 2**), indicating that location, and presumably environmental factors, greatly impact foliar endophyte fungal communities. Therefore, potential differences in the environments of the native habitats of the disjunct pairs in EA and ENA may have also resulted in significant differences in the FEF diversity between them, although this comparison was not made. The potential impacts of endophytes in modifying the fitness of the host species, e.g., by providing protection from microbial pathogens (Arnold et al., 2003) or by reducing herbivore fecundity (Van Bael et al., 2009; González-Teuber, 2016), have been reported. The potential differentiation of foliar endophyte assemblages between the disjunct species in their native habitats, therefore, could have been an important factor driving allopatric differentiation of species’ features in EA and ENA. Our comparison of FEF assemblages in species based on regions of origin (i.e., in all EA species versus those in all ENA species) from the Arnold Arboretum revealed a Bray-Curtis dissimilarity value of 0.53, indicating an intermediate level of dissimilarity in FEF assemblages between the two regions. This observation suggests the possibility of a regional difference in processes that shaped the turnover of FEF communities in EA and ENA, likely because of potential differences in the abiotic environment. However, combined with the result from PCoA that showed no overall clustering of samples and species from EA vs. those from ENA and only 1.4% variation explained by regions of origin (EA or ENA), evidence suggests the regional effect on the leaf endophyte assemblage is likely weak. Future comparisons of ecological niches and FEF communities of host sister species in EA and ENA in their native habitats are necessary to test the hypothesis of environment-driven divergence of FEF communities in disjunct species. Such studies can reveal the specific environmental factors associated with the changes of their FEF assemblages in the diverged species.

Several lines of evidence from our study are consistent with previous studies in supporting important roles of host identity (including individuals/genotypes) in FEF assemblage (Solis et al., 2016; Vincent et al., 2016; Liu et al., 2019). These include great dissimilarities of FEF diversity and composition between species and genera (**Supplementary Table 2**), greater variation of FEF diversity and composition among individuals of the same species (intraspecific variance) than between species from the same sampling locations (**Table 2**), as well as many indicator species from each genus (FEF ASVs restricted to a single genus, **Supplementary Table 3**) with a low number of ASVs shared among genera (**Supplementary Table 4**). The evidence also suggests that FEF community differences is more host-plant and environment-dependent than driven by evolutionary and historical biogeographic isolation (i.e., phylogenetic distance) in these disjunct taxa, and different host plants can impose different habitat filtering in determining the FEF compositions, as previously reported (Saunders et al., 2010).

Previous studies revealed no association between phylogenetic distance of 11 plant species spanning deep phylogenetic divergence (across families) and FEF assemblages (Vincent et al., 2016), but a significant association between these two variables was observed for recently diverged species (i.e., within a single genus *Ficus*; Liu et al., 2019). We observed a significant association between phylogenetic distance and FEF beta diversity among species of 17 genera with EA-ENA disjunct species pairs spanning both shallow (sister species within genus) and deep phylogenetic divergence (major groups of angiosperms and gymnosperms represented by different genera) (r = 0.083, P < 0.001; **Figure 3A**). The significant correlation between phylogenetic distance and dissimilarity of FEF assemblages detected in disjunct species pairs was based on the phylogeny of the host species that reflects much of the backbone of angiosperm phylogeny (Angiosperm Phylogeny Group IV, 2016). Furthermore, the phylogeny was estimated with data from 14 chloroplast genes shared by most species, which minimized the influence of missing data on both phylogeny reconstruction and phylogenetic distance estimation. An association between phylogenetic distance and FEF community dissimilarity supports a role of phylogeny in shaping FEF assemblages, although the role is not significant (r = 0.083, P < 0.001; **Figure 3A**), especially in closely related species. Our results from regression analysis of divergence time (reflecting phylogenetic distance) and dissimilarity of FEF communities indicate that the degree of difference in the FEF community is also not associated with the duration of isolation of the species pairs (**Figure 3C**; r = 0.004; *P* = 0.79) and that many species pairs diverged at similar times but differed in their levels of divergence in the FEF assemblages between the species counterparts. These results suggest a minor role of deep-level host-plant phylogeny in shaping the overall FEF assemblage and a greater effect of certain families, orders, and classes on abundance of some FEF genera at more recent divergences of host species (e.g., among congeneric species). Our results from PCoA and multiple statistical tests of beta diversity with various groupings of host species support host identity and environments as having played major roles in shaping the pattern of FEF variation in the EA-ENA disjunct flora.

We observed no significant association of phylogenetic distance and FEF beta diversity within *Cornus* (**Figure 3B**), which differs from findings in the study of *Ficus* (Liu et al., 2019). Assessment of the role of phylogeny on FEF assemblages critically relies on the robustness of the phylogeny of the host species. Sampling of host species and molecular data both strongly affect the accuracy of estimation of phylogenetic distance, whereas many factors, including variation of habitats of host species, also affect accuracy of estimation of dissimilarity of FEF assemblages in host plants. The species sampling of *Ficus* was from plants grown in the same botanical garden (Xishuangbanna Botanical Garden, China) and the phylogenetic construction used three DNA sequences (ITS, ETS, and one nuclear gene). In our study of *Cornus*, the species sampling was from plants grown together in the Arnold Arboretum, but was heavily biased toward the *Swida* clade (with many more species sampled than from other clades), which contains closely related species (**Figure 1**). This biased sampling may have affected the Mantel test result. However, we detected multiple endophyte taxa (mainly from Ascomycota and Basidiomycota) associated with host phylogenies in both disjunct species and within *Cornus* (**Table 3**). For instance, the beta diversity of species of the fungus *Sphaerulina* (Mycosphaerellaceae) (some of which are known fungal pathogens causing leaf spot and stem cankers - e.g., in poplar, Naik et al., 2021) are significantly correlated (Spearman’s rank in the Mantel test) with the phylogenetic distance among *Cornus* hosts (r = 0.6, **Table 3**). It would be interesting to evaluate the roles of *Sphaerulina* in the ecology (such as disease resistance), diversification, and evolution of *Cornus*. The observation of overall non-significant association between phylogenetic distance and FEF dissimilarity within *Cornus* here and among the 11 distantly related species in Vincent et al. (2016) may be an artifact of host species sampling or data sampling or both; that is, uneven density of host sampling among the three clades of *Cornus* may have led to an excess of closely related species among those sampled, and a diverse and small set of plant taxa might well explain that the molecular markers used could have a saturation of substitution issue that biased phylogenetic distance calculation.

Our study shows that FEF diversity appears to be much higher than FEB diversity in the species we investigated. In several species, we detected fewer than 10 bacterial ASVs (**Supplementary Figure 3**). A lower diversity of FEB than FEF may reflect true differences or could be attributed to several other reasons. First, it may be partly due to differences of fungi and bacteria in their DNA degradation. The procedure of leaf sample preparation used could have resulted in more degradation of bacterial DNA (by DNAse in the cell) or low PCR primer efficiency. Second, it may be due to a difference in the discrimination power of the 16S rDNA and ITS markers. Although the primer pair we used was specific to exclude 16S rDNA from chloroplast genomes, the 16S rDNA region is usually more conserved and less sensitive or powerful for detecting taxon diversity compared to the ITS region that is highly variable among species. Third, the difference in ASVs between bacterial and fungi in each plant species could also be attributed to potential misclassification due to errors in databases; the current bacterial and fungal databases are still being updated. Much lower ASV diversity of FEB compared to FEF was also reported by Wang et al. (2016), while other studies showed higher diversity of FEB than FEF in the same species (Horton et al., 2014; Griffin, 2016; Wemheuer et al., 2019). The patterns of FEB diversity and assemblages observed in our study are largely similar to those observed for FEF in supporting major roles of host identity and environment, but the impact of sampling locations seemed to be smaller (low r values in ANOSIM and low R^2^ values in PERMANOVA) than observed for FEF (**Table 2**).

## 5 Conclusions

In conclusion, our comparative study of foliar endophytes in EA-ENA disjunct species pairs and *Cornus* revealed large differences in foliar endophyte communities among individuals within species, among genera, and among geographic (or sampling) locations, but minor differences between species of the same genus and between EA and ENA. The variation of FEF assemblages observed can be explained mainly by a combination of factors including phylogenetic divergence, historical geographic isolation, sampling locations (environment), and host identity, with the latter two factors being much more important. Our finding of sampling locations playing a major role in shaping the FEF assembly in the disjunct taxa implies there would likely be significant differences of FEF communities in disjunct counterparts in their native habitats in EA and ENA, although this is still untested. The differences may have been important in driving allopatric divergence of functional traits of the disjunct sisters in EA and ENA, a hypothesis that can be tested in the future. To our knowledge, this is the first foliar endophyte analysis of disjunct species pairs in EA and ENA across a diverse array of lineages. The results provide a new perspective on the post-isolation evolution of disjunct taxa of the prominent EA-ENA floristic disjunction and contribute to better understanding of the relationship between the diversity and assemblage of endophytes and the phylogeny of their host plants.

## Data availability statement

The datasets presented in this study can be found in online repositories. The names of the repository/repositories and accession number(s) can be found in the article/**Supplementary Material**.

## Author contributions

WZ: Data curation, Formal Analysis, Funding acquisition, Investigation, Methodology, Visualization, Writing – original draft, Writing – review & editing. WS: Formal Analysis, Methodology, Software, Writing – review & editing, Funding acquisition, Supervision. PS: Supervision, Writing – review & editing, Funding acquisition. DS: Supervision, Writing – review & editing, Funding acquisition. QX: Funding acquisition, Project administration, Resources, Supervision, Validation, Writing – review & editing, Conceptualization.
