# Supplementary material for: Foliar endophyte diversity in Eastern Asian-Eastern North American disjunct tree species – influences of host identity, environment, phylogeny, and geographic isolation
Source: Front Plant Sci. 2023 Dec 13;14:1274746. doi: 10.3389/fpls.2023.1274746 (PMC10773735; doi:10.3389/fpls.2023.1274746)
Supplement: Supplementary file 1 [file Image_1.pdf]

Supplementary Material

1 Supplementary Figures and Tables

1.1 Supplementary Figures

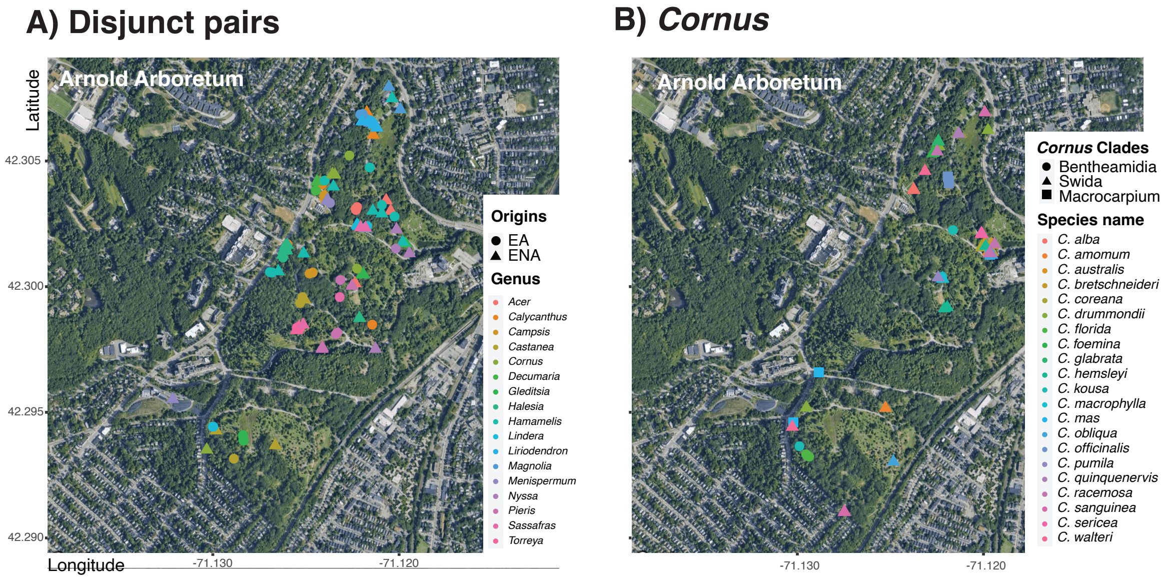

Supplementary Figure 1. A) Collecting map of all disjunct pair species from Arnold Arboretum. B) Collecting map of all *Cornus* species from Arnold Arboretum.

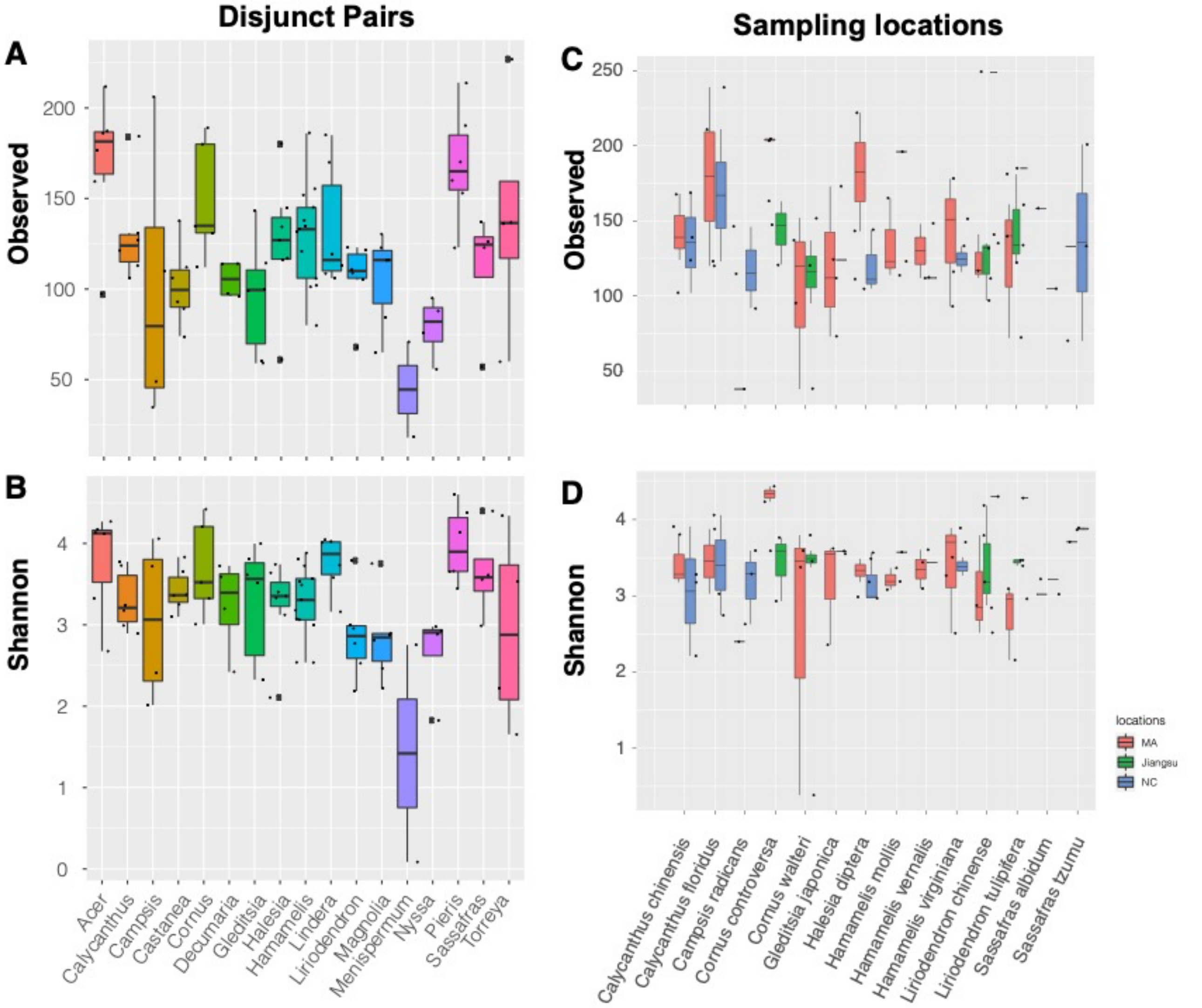

Supplementary Figure 2. Foliar endophytic fungal alpha diversity of 17 disjunct genera and species from different sampling locations including A) Observed ASVs and B) Shannon Index of 17 disjunct genera; C) Observed ASVs and D) Shannon Index of species from different geographic locations (Boston, MA, USA: red; Raleigh and Durham, NC, USA: blue; and Nanjing, Jiangsu, China: green).

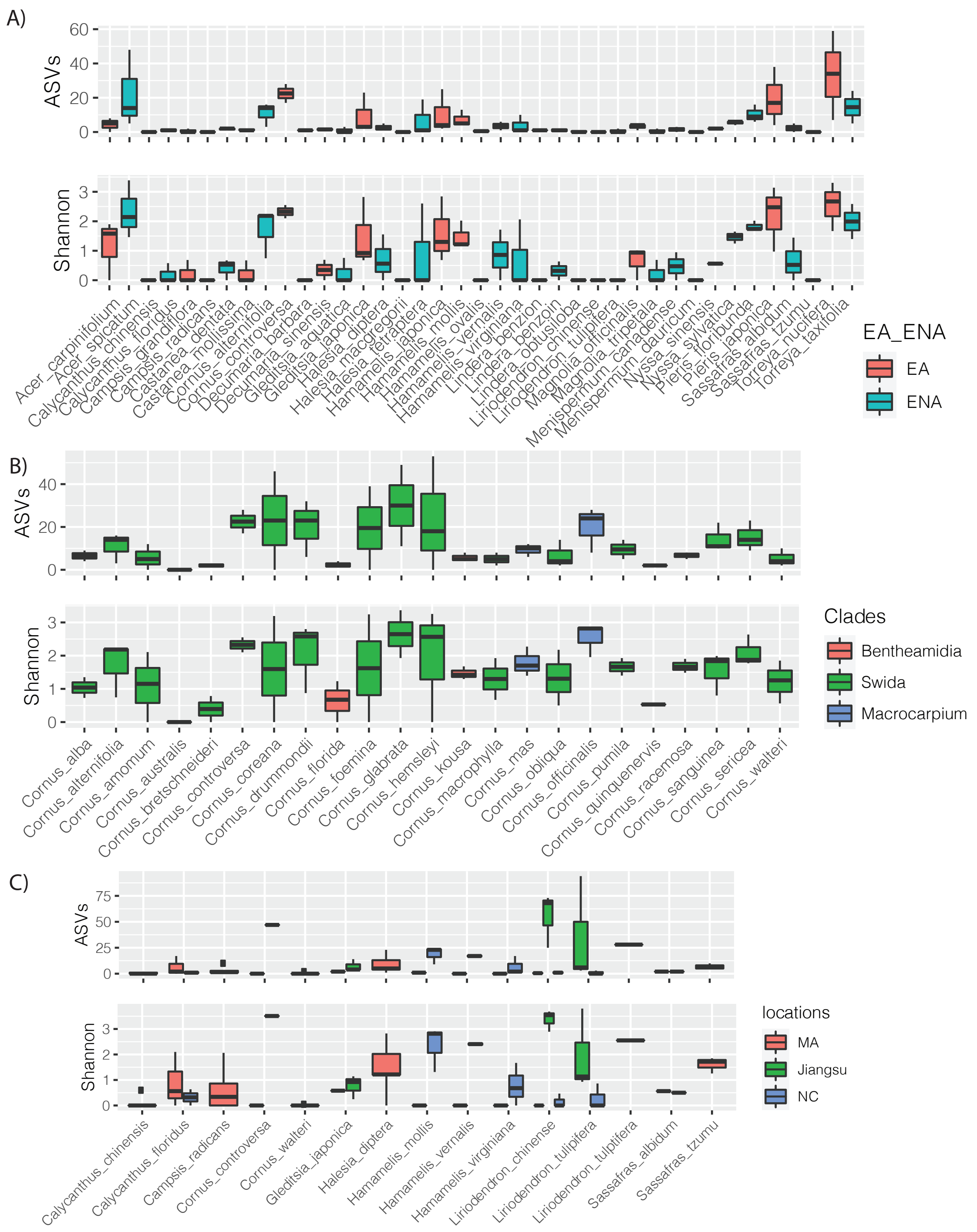

Supplementary Figure 3. Foliar endophytic bacteria alpha diversity ASVs including Observed ASVs and Shannon Index A) 17 disjunct pairs (red color represents EA; blue color represents ENA), B) 23 *Cornus* species (red color represents Benthamidia clade; green color represents Swida clade; while blue color represents Macrocarpium clade), and C) 14 genera from three different locations (Boston, MA, USA: red; Raleigh and Durham, NC, USA: blue; and Nanjing, Jiangsu, China: green).

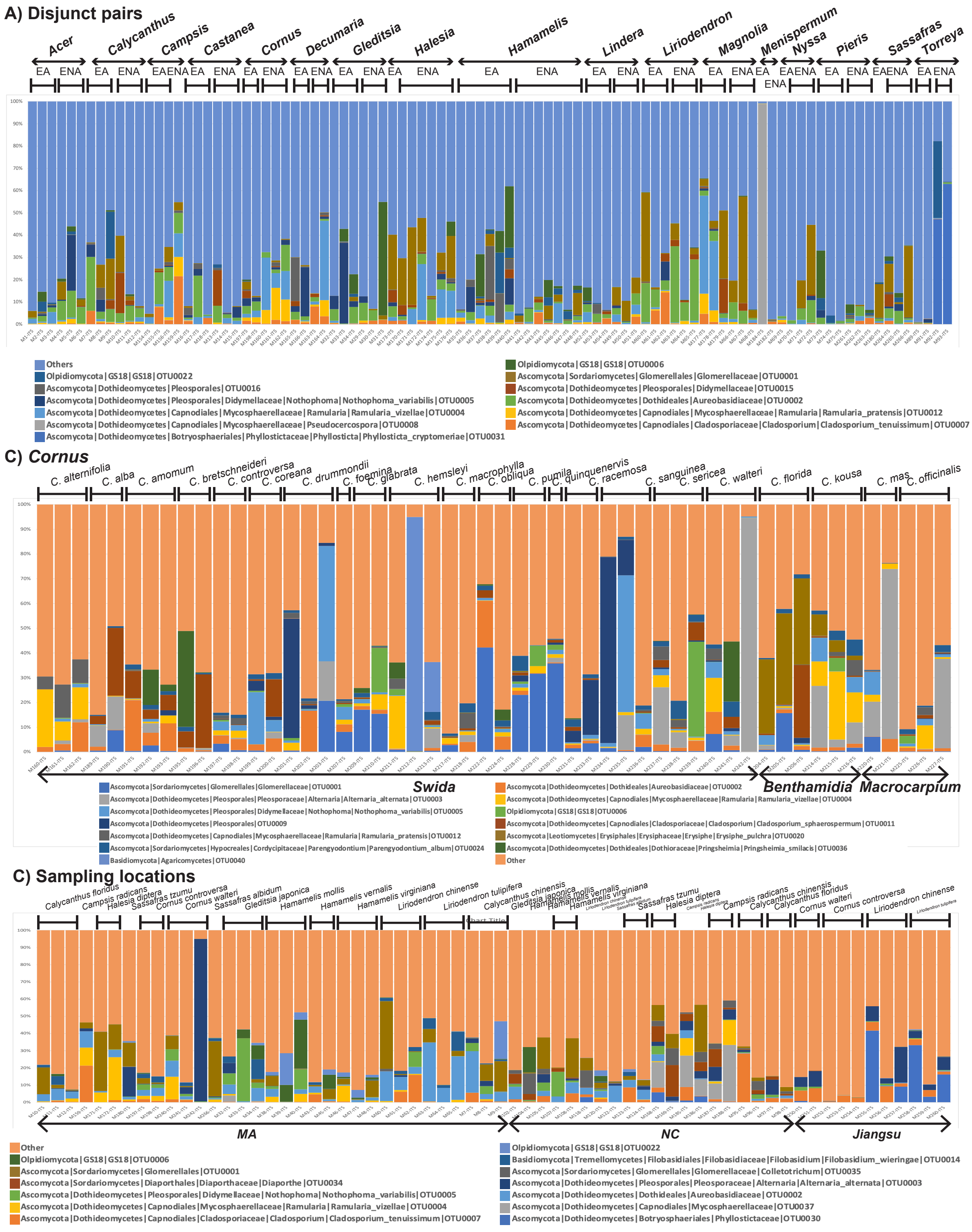

Supplementary Figure 4. Relative abundance of major (dominant) fungal ASVs with >1% for A) disjunct pair species, B) *Cornus* species, and C) species from three geographic/sampling locations. The plot was for individuals. Only ASVs with these fungal abundance >1% were plotted. The fungal ASVs are color coded. The individuals included in the analysis are coded as M#-ITS on the X-axis (Supplementary Table 1).

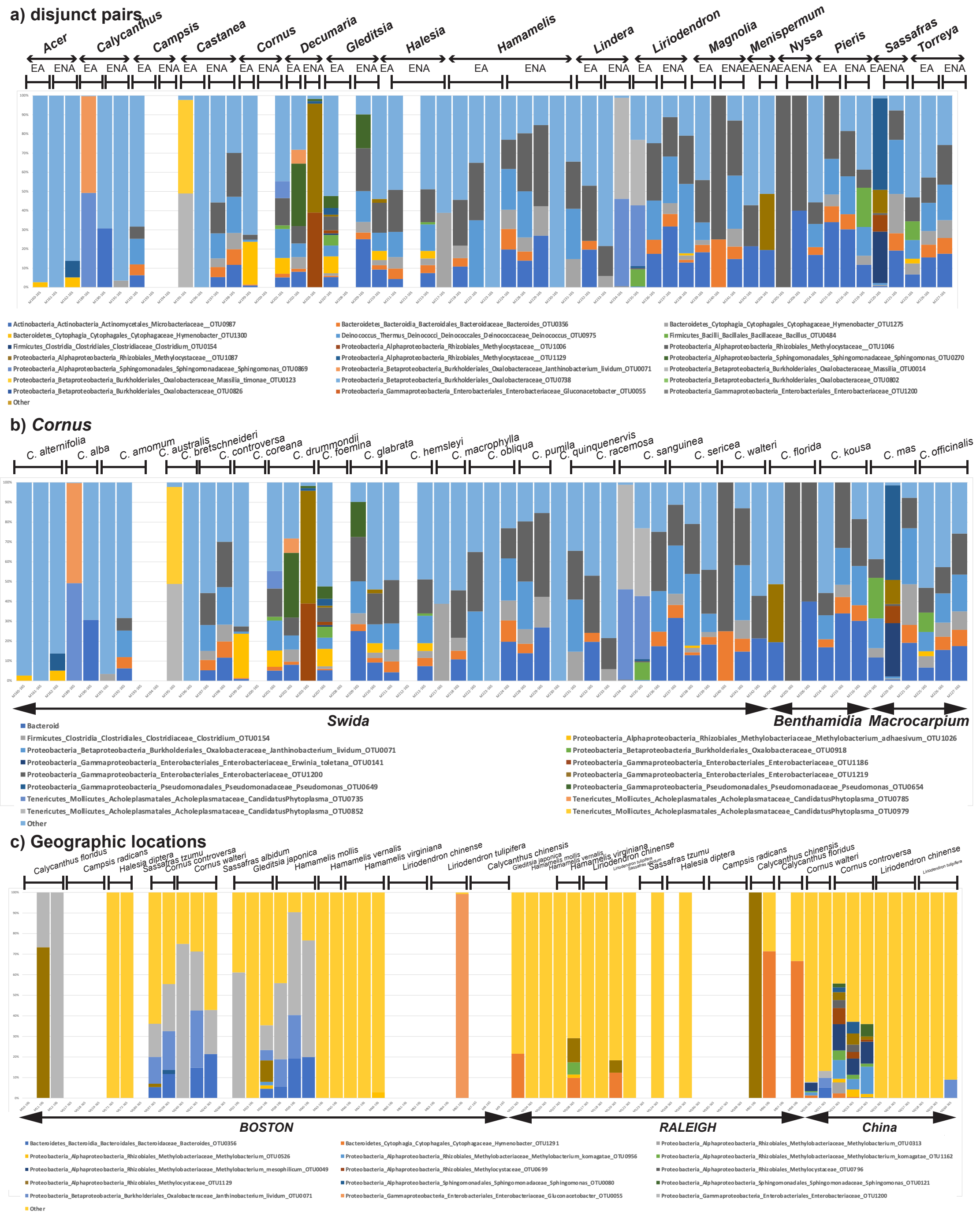

Supplementary Figure 5. Relative abundance of major bacterial ASVs for A) disjunct pair species, B) *Cornus* species, and C) species from three geographic/sampling locations. The plot was for individuals. Only ASVs with abundance over 1% were plotted. The bacterial ASVs are color coded. The individuals included in the analysis are coded as M#-16S on the X-axis (Supplementary Table 1).

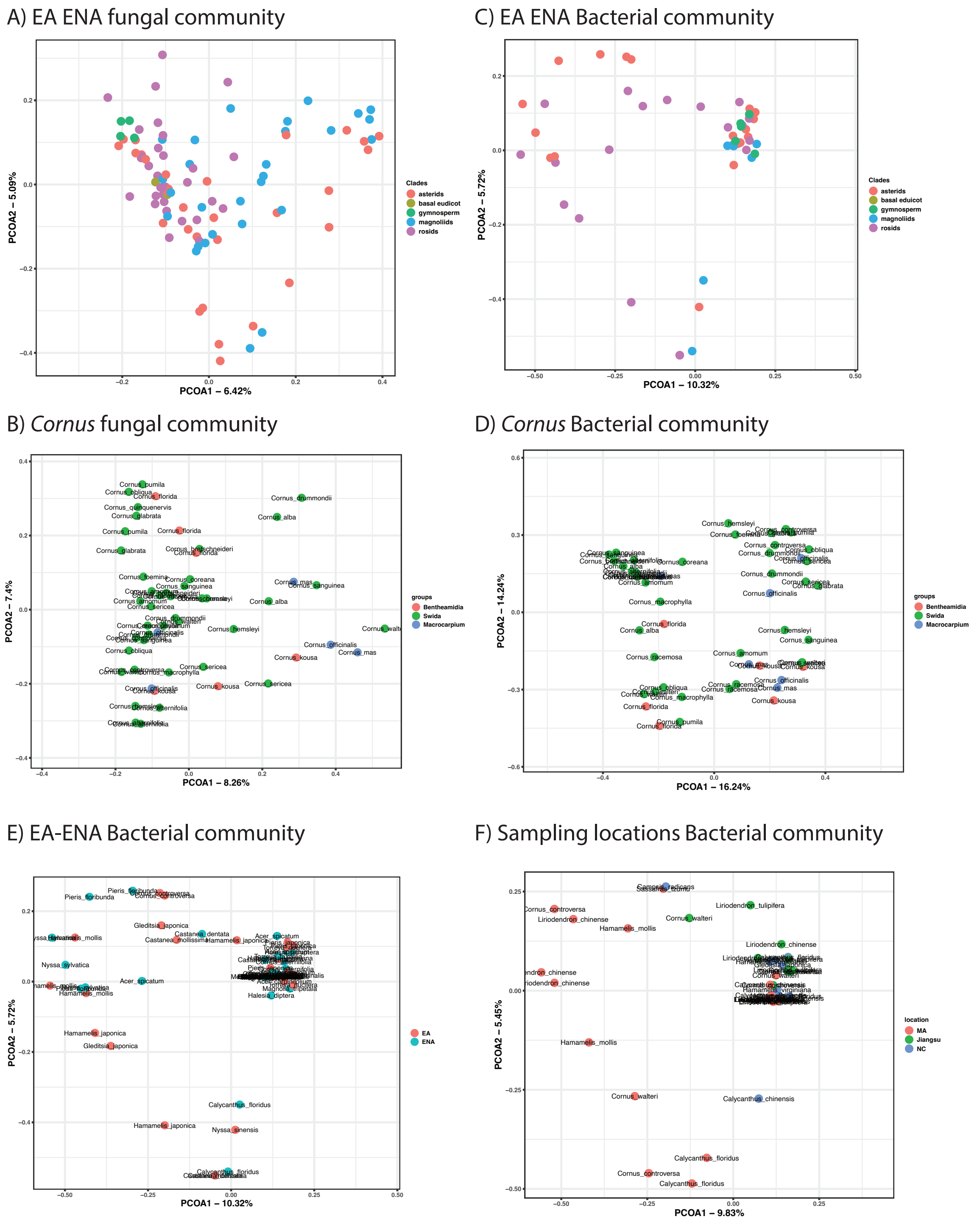

Supplementary Figure 6. Principal-coordinate analyses (PCoA) plots of Bray-Curtis ITS profiles. A) 17 disjunct pairs of EA-ENA counterparts (fungi). The scatter plot is colored by five different clades, including asterids, basal eudicot, gymnosperm, magnoliids, and rosids (including super-rosids) (ANOSIM, *P* = 0.001; PERMANOVA, *P* = 0.001 in Table 2), B) *Cornus* species (fungi). The scatter plot is colored by three different clades, including BB, BW, and CC clades (ANOSIM, *P* = 0.386; PERMANOVA, *P* = 0.001 in Table 2). C) 17 disjunct pairs of EA-ENA counterparts (bacteria). D) *Cornus* species (bacteria). E) 17 disjunct pairs of EA-ENA counterparts (bacteria). The

scatter plot is colored by EA and ENA. F) three different geographic/sampling locations (bacteria). The scatter plot is colored by three locations: Boston, MA, USA; Raleigh and Durham, NC, USA; and Nanjing, Jiangsu, China.
